# Perforin-dependent direct cytotoxicity in natural killer cells induces considerable knockdown of spontaneous lung metastases and computer modelling-proven tumor cell dormancy in a HT29 human colon cancer xenograft mouse model

**DOI:** 10.1186/1476-4598-13-244

**Published:** 2014-11-05

**Authors:** Tobias Brodbeck, Nina Nehmann, Anja Bethge, Gero Wedemann, Udo Schumacher

**Affiliations:** Experimental Morphology, Center for Experimental Medicine, University Medical Center Hamburg, Martinistrasse 52, 20246 Hamburg, Germany; Competence Center Bioinformatics, Institute for Applied Computer Science, University of Applied Sciences Stralsund, Zur Schwedenschanze 15, 18435 Stralsund, Germany

**Keywords:** Natural killer cell, Perforin-dependent direct cytotoxicity, Spontaneous lung metastases xenograft, HT29 colon cancer cell line, Computer modelling

## Abstract

**Background:**

For long, natural killer (NK) cells have been suspected to play a critical role in suppressing the development of spontaneous metastases in cancer patients. Despite a wide range of studies it remains unclear so far to what extent primary tumor growth together with formation of distant metastases and NK cell activity influence each other.

**Methods:**

To precisely investigate the role of NK cells with a perforin-deficiency in cancer growth and metastasis formation, human HT29 colon cancer cells were subcutaneously grafted into pore forming protein and recombination activating gene 2 double knock out (pfp/rag2) mice and in recombination activating gene 2 only knock out (rag2) mice both with black six background. Both mice lack B and T cell functions due to the absence of rag2.

**Results:**

Primary tumors developed in 16/16 in pfp/rag2 and 20/20 rag2 mice. At sacrifice primary tumor weight did not differ significantly. However, tumors grew faster in pfp/rag2 mice (50 days) than in pfp/rag2 mice (70 days). Circulating tumor cells (CTC) in murine blood were nearly three times higher in pfp/rag2 (68 cells/ml) than in rag2 mice (24 cells/ml). Lung metastases occurred frequently in pfp/rag2 mice (13/16) and infrequently in rag2 mice (5/20). The mean number of metastases was 789 in pfp/rag2 mice compared to 210 in rag2 mice. Lung metastases in pfp/rag2 mice consisted of 10–100 tumor cells while those in rag2 mice were generally disseminated tumor cells (DTCs).

Computer modelling showed that perforin-dependent killing of NK cells decelerates the growth of the primary tumour and kills 80% of CTCs. Furthermore, perforin-mediated cytotoxicity hampers the proliferation of the malignant cells in host tissue forcing them to stay dormant for at least 30 days.

**Conclusion:**

The results exactly quantified the effect of perforin-dependent direct cytotoxicity of NK cells on HT29 on primary tumor growth, number of CTCs in the blood and the number of metastases. The largest effects were seen in the number of mice developing spontaneous lung metastases and the mean number of lung metastases. Hence, perforin-mediated cytotoxicity used for direct killing by NK cells is more important than indirect killing by secretion of death-inducing ligands by NK cells.

## Background

In the developed world cancer represents the second most common cause of death and more than 90% of these deaths can be attributed to its metastatic spread [1–3]. Therefore control of the metastatic process is the key determinant to an improved survival of cancer patients. The control of cancer cells in the body is complex but one cell population, natural killer (NK) cells, represents the prime position in the defense against cancer cells as it can kill malignant cells without the necessity of prior exposure [4, 5].

NK cells constitute a heterogeneous population of large granular lymphocytes that comprise approximately 10-15% of peripheral blood mononuclear cells (PBMC) in humans. Belonging to the innate immune system NK cells are considered as lymphocytes capable to discover and destroy tumor and virus-infected cells [6]. The name natural killer cells has its origins in their ability to kill virus infected cells spontaneously without the necessity for priming as generally required in the specific immune system [7–9].

Their phenotype is characterized by the surface expression of CD56 (NCAM) and/or CD16 as well as the lack of expression of CD3 [8, 10]. In comparison to T cells NK cells do not require the presence of antibodies and surface antigens stringently for their activation. Furthermore they have the unique skill to recognize and respond to the absence of MHC class I antigens on the target cell which is an immunoescape mechanism frequently used by tumor and virus-infected cells to avoid CD8 T-Cell mediated lysis [11–13]. Once activated NK cells have a multitude of mechanisms at their disposal to attack neoplastic cells. One of this mechanisms is the direct release of cytolytic granules containing perforin, granzymes and granulysin by exocytosis to kill target cancer cells [14, 15].

Because of their anti-cancer cell activity a wide range of studies postulates relationships between decreased NK-cell activity and advanced tumor growth as well as the formation of distant metastases but it remains unclear so far to what extent primary tumor growth together with formation of distant metastases and NK cell activity influence each other. Numerous studies indicate that metastatic spread is associated with decreased NK cell activity [16–19] and clinical observations suggest NK-cell activity to be a strong predictor for overall and progression-free survival in carcinoma patients [20, 21]. These clinical findings may be given support by a xenograft mouse model where it could be shown that in small cell lung cancer (SCLC) metastatic spread increased drastically when perforin-dependent killing was disabled in NK cells [22]. However, the two mouse strains used in this experiment had disparate genetic backgrounds. Since the genetic background can significantly impact tumor development [23, 24] the crucial role of NK cells on metastatic spread has not been able to clearly demonstrate as yet.

In order to study the influence of perforin-dependent direct cytotoxicity of NK cells on primary tumor growth and the number of metastatic deposits formed, we used two different mouse strains, namely rag2 and pfp/rag2 mice on the same genetic background. Both mouse strains lack the recombination activating gene (rag2) and therefore share the inability to produce functioning T- and B-cell receptors by V(D)J-recombination. The special characteristic of the pfp/rag2 mice is the additional absence of the pore forming protein (pfp) leading to a suppression of the cytotoxicity of NK cells. The crucial advantage of this xenograft mouse model in comparison to other models investigating the mechanisms of metastatic spread is the fact that these two mouse strains have a completely identical genetic background and are only different in the pfp knock out [25–28]. This direct approach is superior to the use of an antibody against NK cells as the antigen recognized on NK cells by the anti-NK cell antibody is also expressed on tumor cells in general [22] and especially also expressed on HT29 cells [29]. Because of this antibody cross-reactivity we used this knock out model.

Furthermore, the results of the animal experiments will be analyzed via computer simulations. Computer models are valuable tools to quantitatively examine the observed results and to offer various explanations for different scenarios of the metastatic process. Thus, they allow additional valuable perspectives in details of the metastatic process hitherto unexpected.

## Results

### Engraftment of primary tumors

**Figure 1:**
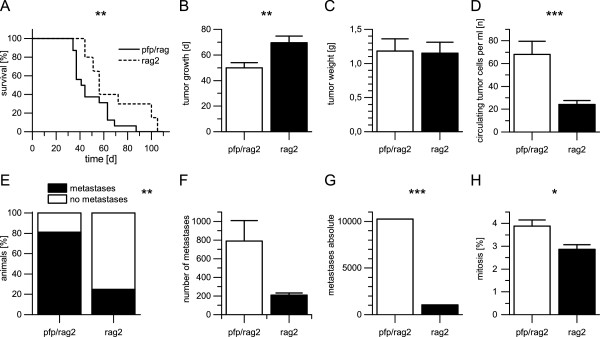
**Overview of the results of the experiment. A)** Pfp/rag2 mice with perforin-deficient NK cells reached termination criteria earlier than rag2 mice due to amplified primary tumor growth and development of ten times more spontaneous lung metastasis (p = 0.0004). **B)** Primary tumors with comparable weight developed after a mean growth period of 69.4 days in rag2 mice compared to only 49.9 days mean growth period in pfp/rag2 mice (p = 0.0058). **C)** The mean weight of primary tumors was 1.16 g in rag mice and 1.23 g in pfp/rag2 mice, thus being statistically not different (p = 0.741). **D)** The mean number of circulating tumor cells detected in murine blood using qRT-PCR was 24 cells per ml in rag2 mice in contrast to 68 cells per ml in pfp/rag2 mice (p = 0.0004). **E)** Only 25% of rag2 mice developed spontaneous lung metastases while 81% of pfp/rag2 mice exhibited spontaneous lung metastases. Perforin-dependent killing inhibited metastatic spread relatively by 56% and by 75% in total (p = 0.002). **F)** In rag2 the mean number of metastases was 210 (n = 5) compared to 789 metastases in pfp/rag2 (n = 13) (p = 0.0629). **G)** The total amount of spontaneous lung metastases in rag2 mice was 1048 compared to 10251 in pfp/rag2 mice (p = 0.0001). Decreased NK cell cytotoxicity led to the development of ten times more spontaneous lung metastases. **H)** In pfp/rag2 3.9% of the tumor cells were in a mitotic state compared to 2.9% of the tumor cells in rag2 (p = 0.0107). Error bars display the standard error of the mean (SEM). Asterisks represent significant differences between both groups (* - p ≤0.05, ** - p ≤0.01 and *** - p ≤0.001). A Mann–Whitney U test was used to calculate statistical significances between both samples.

Overall tumors developed in 100% (n = 16) of the pfp/rag2 mice and in 100% (n = 20) of the rag2 mice. The duration of the experiment ranged from 45 to 106 days in the rag2 mice while it ranged from 35 to 88 days in the pfp/rag2 mice. The mean growth period in the rag2 mice was 69.4 days compared to 49.9 days in the pfp/rag2 mice (Figure 1A, B). The tumor weight range of the rag2 mice varied between 0.08 g and 2.61 g (mean 1.16 g) whereas in the pfp/rag2 mice the weight range varied between 0.20 g and 2.74 g (mean 1.23 g) (Figure 1C). Variances were not statistically significantly different (p = 0.7353).

### Circulating tumor cells in blood

The number of circulating tumor cells detected in murine blood by using qRT-PCR varied between 0 and 124 cells per ml (mean 24 cells per ml) in the rag2 mice compared to the pfp/rag2 mice where numbers varied between 1 and 368 cells per ml (mean 68 cells per ml) (Figure 1D). Variances were statistically significantly different (p = 0.0004).

### Number of spontaneous lung metastasis

25% of the rag2 mice engrafted with HT29 cells (n = 20) developed spontaneous lung metastases while 81% of pfp/rag2 mice equally engrafted with HT29 cells (n = 16) developed spontaneous lung metastasis detected in HE stained sections (Figure 1E). Within the five rag2 mice that had developed spontaneous lung metastasis the number of metastasis varied between 132 and 264 (n = 5, mean 210). The number of spontaneous lung metastasis in 13 pfp/rag2 mice where metastatic spread of the primary tumors had taken place varied between 67 and 2990 (n = 13, mean 789) (Figure 1F). In total 1048 spontaneous lung metastases were detected in rag2 mice while 10251 spontaneous lung metastases were detectable in pfp/rag2 mice (Figure 1G). Variances were statistically significantly different (p = 0.0001).

### Morphology

**Figure 2:**
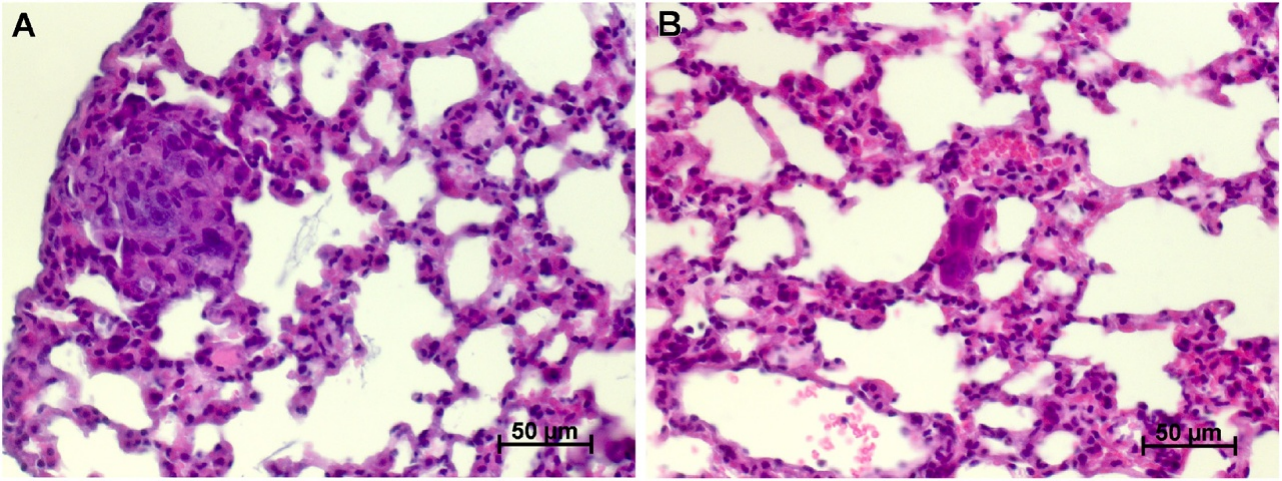
**Typical metastases in pfg** **/** **rag2 and rag2 mice. A)** Typical metastasis of pfp/rag 2 mice with 10–100 malignant cells forming the metastatic deposit. **B)** Typical metastasis of rag2 mice with only very few malignant cells. Magnification: 400X, staining method: haematoxylin and eosin (H.E.).

The metastasis detected in the two different mouse strains did not only differ in their number but also in their morphology. Typical metastasis detected in rag2 mice consisted of only 1–10 cells at the maximum whereas disseminated tumor cells (DTC) were the most common presentation of metastasis in the rag2 mice. All cells were clearly extravasated. In pfp/rag2 mice typical metastases consisted of approximately 10–100 tumor cells and only sparse DTCs occurred (see Figure 2A, B)

### Feulgen staining

In rag2 mice 2,9% of the tumor cells were mitotic compared to 3.9% of the tumor cells in pfp/rag2 mice (Figure 1H). Variances were statistically significantly different (p = 0.0107).

### Computer modelling of cancer spread

In addition to the sole statistical analysis of the data the experimental data was further analyzed utilizing a computer model of the process of the metastatic progression [30, 31]. This computer model allows simulating various different scenarios that can be compared with the experimental data providing a quantitative analysis of the processes underlying metastatic progression in order to generate new hypotheses.

Using the a gompertzian growth function (see equation (1) in the Methods section) and the observed mean values for the tumor weight and the duration of the experiment (1.23 g and 49.9 days for pfp/rag2; 1.16 g and 69.4 days for rag2 mice) a growth rate constant of 0.0462 day^-1^ was determined for pfp/rag2 mice and 0.0326 day^-1^ for rag2 mice.

The spread of metastases was modelled via a colonization rate (see equation (2) in the Methods section). Based on the number of metastases in the lung (788 after 49.9 days in pfp/rag2) a colonization coefficient of 5.2e^-5^ (cell*day)^-1^ was computed for pfp/rag2. Since the same cell type was used in both mouse types, the primary tumor should exhibit the same spreading behavior in the pfp/rag2 and the rag2 mouse stain. Therefore, the determined colonization coefficient for pfp/rag2 mice was also applied for rag2 mice. To comprise the lesser amount of metastases in the rag2 mouse stain (209 after 69.4 days), the mortality of cycling tumor cells was increased. Different values were tested in the computer model, but only a mortality rate of 80% resulted in the observed number of metastases for rag2 mice, implicating that 80% of those cells that would have been able to found a metastasis in pfp/rag2 mice are killed by NK cells in rag2 mice.

**Figure 3:**
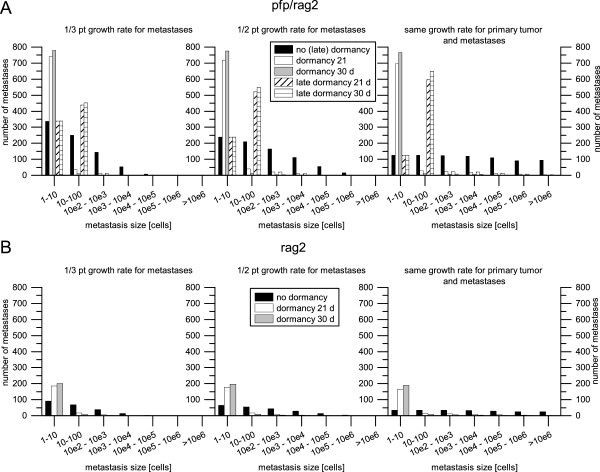
**The number of lung metastasis in different size ranges for pfp** **/** **rag2 mice (A) and rag2 mice (B).** The metastases size ranges are scaled logarithmically. Three different growth rates were applied on the lung metastases: 1/3 (left), 1/2 (middle) and same (right) growth rate as the primary tumor. According to the observed morphology, metastases in the rag2 mouse stain **(B)** remain dormant for at least 30 days, before they are able to successfully proliferate. In pfp/rag2 mouse strain **(A)** metastases are able to successfully proliferate much easier. However they undergo a late dormancy, once they already proliferated a few times and reached a size between 10 and 100 cells.

At distant sites NK cells hamper the establishment of DTCs into a metastasis (see Figure 3). Metastases in pfp/rag2 mice have approximately 10–100 cells while in rag2 mice mostly DTCs were observed. The simulation results where no sort of dormancy was applied (black bars in Figure 3) do not fit with the observed morphology of the metastases in pfp/rag2 mice and rag2 mice.

Disseminated tumor cells in rag2 mice remain dormant for at least 30 days, before they are able to successfully proliferate (grey bars in Figure 3B). Applying shorter dormancy time spans, e.g. 21 days (white bars in Figure 3B), several metastases comprising more than 10 cells occur. Simulations with a dormancy of 30 days resulted in mostly disseminated tumor cells and only few multicellular metastases. The different growth rates applied on the metastases (1/3, 1/2 and the same growth rate as the primary tumor) had nearly no effect on the simulation results, as can be seen in Figure 3B.

In pfp/rag2 mice neither the simulation results with no dormancy (black bars in Figure 3A) nor the simulation results with a dormancy of 21 and 30 days (white and grey bars in Figure 3A) fit with the observed morphology. Therefore, a late dormancy was introduced: After the metastasis reaches a random size between 10 and 100 cells it stops growing for a certain time span. An explanation could be that the metastasis has to initiate neoangiogenesis to ensure nutrition and oxygen supply for further growth. The simulation results with an applied late dormancy of 30 days and a metastases growth rate the same as the primary tumors (cross-striped bars in the rightmost graph in Figure 3A), fit quite well with the observed morphology, indicating that the metastases in pfp/rag2 grow at the same rate as the primary tumor.

Summarizing, according to results of the computer simulations, NK cells decelerate the growth of the primary tumor and kill 80% of those cycling tumor cells which could have otherwise established a new metastasis. Furthermore, they hamper the proliferation of the malignant cells in distant tissue forcing them to stay dormant for at least 30 days. Without NK cells it is much easier for malignant cells to establish themselves in the distant tissue and start proliferating. However, they also undergo a dormant phase of at least 30 days after they reach a size between 10 and 100 cells.

## Discussion

The major aim of this study was to investigate to which extent perforin-dependent direct killing of natural killer cells influences both the growth of a primary tumor and the formation of metastases in vivo by using a HT29 xenograft mouse model. Experimental findings that natural killer cells might play a crucial role in the development of primary tumors as well as in spontaneous metastases formation have been described in earlier studies although their exact mode of action has remained elusive so far [9, 32–34]. In an earlier xenograft model using scid and pfp/rag2 mice it has been shown that the number of spontaneous SCLC metastasis increased when perforin-mediated direct cytotoxicity of NK cells was disabled [22]. However the genetic backgrounds of the used mouse strains were not identical as scid mouse in this study had a Balb/cBy background whereas the background of the pfp/rag2 mouse strain was C57BL/6 J.

In order to exclude this strain difference as a possible reason for the difference in metastasis formation and to examine whether natural killer cells and perforin-dependent direct cytotoxicity in particular do play a crucial role in the growth of a primary tumor and its spread to distinct organs we used pfp/rag2 and rag2 mice of the same genetic background to investigate the importance of perforin-dependent killing by NK cells upon primary tumor growth and formation of distant metastasis in vivo.

The average survival of the pfp/rag2 mice was shortened to 49.9 days compared to 69.4 days in the rag2 mice as the mice had reached the termination criteria. This improved survival rate is validated by clinical data showing that colorectal cancer patients with extensive NK cell infiltration had a 5-year cumulative survival rate of 92% compared to only 53.7% of patients with little or moderate NK cell infiltration of their tumors [35]. The mean weight of the primary tumors was nearly identical in both mouse strains with differences in tumor weight being not statistically significant. The primary tumors of pfp/rag2 mice grew to a similar weight as the primary tumors of rag2 mice in only 72% of rag2 mice’s average lifetime. Thus the diminished direct NK cell cytotoxicity apparently enables primary tumors to grow considerably faster even if NK cells were only scarce in colorectal cancer tissue from early stages on as demonstrated in clinical studies [36]. Feulgen immunohistostaining also revealed that primary tumors of pfp/rag2 mice additionally showed statistically significantly more mitotic cells than primary tumors of rag2 mice. Hence direct killing of NK cells considerably influences primary tumor growth in immunodeficient mice.

Furthermore the 2.8 times higher number of circulating tumor cells in murine blood of pfp/rag2 mice compared to rag2 mice indicates that perforin-dependent killing of NK cells is very important for the elimination of circulating tumor cells in murine blood in order to prevent the formation of spontaneous metastases in distant organs. This is in line with the data generated by a computer modelling of cancer spread which revealed that 80% of the circulating tumor cells in the blood of rag2 mice had been eliminated by NK cells before extravasation was possible. These observations are corroborated by clinical findings showing that SCLC patients with high NK cell numbers in the blood had a relatively better prognosis with less distant metastasis than those patients with fewer NK cell numbers [37].

Unsurprisingly direct cytotoxicity of NK cells does have a major effect on the formation of spontaneous lung metastases as only 25% (n = 20) of the rag2 mice exhibited metastatic spread into the lung at all and the presence of NK cells completely prevented the formation of spontaneous lung metastasis in 75% of the rag2 mice. In contrast 81% (n = 16) of pfp/rag2 mice presented spontaneous lung metastases and only 3 animals, 19%, did not develop lung metastases. The absolute number of spontaneous metastases detected in pfp/rag2 mice is 9.78 times higher than the number of spontaneous metastases of rag2 mice. The almost ten times higher numbers of spontaneous lung metastasis found in pfp/rag2 mice in comparison to 3 times higher numbers of circulating tumor cells in the bloodstream of pfp/rag2 mice indicate that perforin dependent direct cytotoxicity of NK cells reduces the number of malignant cells significantly in both the bloodstream and the connective tissue stroma in the distant organ.

In addition to the number of spontaneous metastases the morphology of the spontaneous lung metastases also differed considerably: metastases in rag2 mice were only 1–10 tumor cells in line with single cell metastases (DTCs) being the most frequent detected metastatic deposits while metastases in pfp/rag2 mice generally consisted of 10–100 tumor cells on average presumably showing that metastasis cell killing also takes place in the metastatic organ. It can be excluded that the observed DTCs in rag2 originate from the initial injection of one million tumor cells, since only five from 20 animals developed DTCs in the lung. Otherwise, all mice should have displayed DTCs in the lung since all mice were injected equally by the same person.

The results of the computer simulations lead to the assumption that direct perforin-mediated killing of NK cells hampers the establishment of metastases in the lung, forcing the malignant cells to stay dormant for at least 30 days. In contrast, when perforin-mediated killing of NK cells is suppressed tumor cells proliferate much easier in the distant tissue. However, after the metastases reaches a size between 10 and 100 cells they also seem to undergo a dormant phase of at least 30 days. This result is unexpected and we do not have an evidence based interpretation why this dormancy occurs, however, this result shows that computer analyses can identify critical points in the growth and development of metastases previously overlooked by less sophisticated statistical analyses.

Considering the fact that the rag2 mice lived 30% longer than the pfp/rag2 mice to develop spontaneous metastasis the difference between the discovered number of metastasis in pfp/rag2 and rag2 mice becomes even more impressive.

Our findings are very similar to the observations made by Sodeur et al. [22] where most notably the total number of metastases of all cell lines used in their experiment were considerably higher in pfp/rag2 mice than in scid mice which leaves hardly any doubt that perforin-dependent direct cytotoxicity of NK cells plays a crucial role in the anti-cancer control of our immune system indicating that the presence of NK cells with the abilitiy of direct perforin-dependent killing is more important in determining the number of metastases than the mouse strain background.

There is clinical uncertainty to what extent tumor growth and NK-cell-activity influence each other. Our results show that perforin-dependent direct cytotoxicity of NK-cells is a major component for combatting tumors and the down regulation of perforin-dependent direct cytotoxicity of NK-cells may lead to amplified primary tumor growth, facilitated earlier dissemination of tumor cells in the bloodstream and the formation of spontaneous distant metastases in particular. Hence the lack of NK-cells enables tumors to spread more easily and expansively.

The enormous differences in the formation of spontaneous metastasis and the dissemination in the bloodstream show that perforin-dependent killing is the major factor of NK cell cytotoxicity in this model as perforin-dependent killing reduced the number of spontaneous metastases by 90% in total.

Other NK cell factors such as linking to death-inducing ligands like FasL and TNF-related apoptosis-inducing ligand TRAIL [14, 15, 38–42] are of minor importance in terms of cancer control.

## Conclusion

Altogether our results can confirm the hypothesis that NK cells and in particular their perforin-dependent direct cytotoxicity play a very important role in preventing metastasis formation as they are able to reduce the number of cycling tumor cells in the blood and prevent the formation of distant metastasis to a high degree. Furthermore the mathematical treatment of the data generated in our mouse model has allowed the discovery that NK cells are able to restrict metastatic proliferation by inducing dormancy of malignant cells which is necessary to further explore and may yield an improved treatment of cancer patients in the future.

## Methods

### Animals and experiments

Sixteen eight week old pfp/rag2 mice and twenty eight week old rag2 mice were used for this experiment. The mice were C57BL6 (pfp/rag-2) mice obtained from Taconic, Hudson, NY (# 001177-MM; B6.129S6-Pfp(tm1)Cirk-RAG2(tm1)Fwa, N12). Both strains of mice were kept under pathogen-free conditions in individually ventilated cages (IVC-Rack, Techniplast Germany) and were fed with sterile standard food (ssniff, Soest, Germany) and water ad libitum. The animals were killed when the tumors started to ulcerate or when the tumor weight exceeded 20% of the original mouse body weight at the beginning of the experiment [43].

The experiment was supervised by the institutional animal welfare officer and approved by the local licensing authority (Behörde für Soziales, Gesundheit, Familie, Verbraucherschutz; Amt für Gesundheit und Verbraucherschutz, Hamburg, Germany, project No. G 09/58).

### Cell culture

The human colon adenocarcinoma cell line HT29 was purchased from the European Cell Culture Collection (Porton Down, Wiltshire, UK). Cells were grown in RPMI 1690 – L-Glutamine (GIBCO, Invitrogen Corp., Grand Island, NY) medium supplemented with 10% fetal calf serum, 1% penicillin and streptomycin and cultured in a humidified atmosphere of 37°C and 5% carbon dioxide.

Each mouse was injected subcutaneously with one million viable tumor cells suspended in 200 μl culture medium RPMI 1690 + L-Glutamine (GIBCO, Invitrogen Corp., Grand Island, NY) between the scapulae.

### Histology

After sacrificing the animals primary tumors were excised and fixed in 4% buffered formaldehyde for 24 h and rinsed with phosphate buffered saline. The tissues were then dehydrated in a series of graded ethanol and embedded in paraffin wax. Five μm thick sections were cut and stained with haematoxylin and eosin (H.E.).

In order to achieve a random distribution of the lung, the lungs were fixed en bloc and were sectioned after fixation into one mm thick lung slices. The slices were placed in warm agar and pressed down with a glass piston. After hardening of the agar the lung slices were processed to paraffin wax.

The agar blocks containing the lung slices were sectioned into five μm thick sections. The total number of sections of each lung was noted. In addition to every 10^th^ section, two series of serial sections (n = 30) out of the middle of the paraffin wax block were preserved for further immunohistological evaluation. Ten of the 10^th^ sections containing the most lung pieces of each wax block were H.E. stained. Metastases were counted in each of the ten stained sections under a microscope (Zeiss, Axioplan2). The number of metastases of each mouse was calculated by using the following term (Mean number of metastasis * total number of sections – 20%), according to an earlier established formula [44].

### DNA extraction from murine blood and cultured human tumor cells

Blood samples were withdrawn from each mouse by puncturing the heart after deep general anesthesia with CO_2_. Approximately 1 ml of blood could be extracted from each mouse and a total of 200 μl mouse blood was prepared for DNA extraction using the High Pure PCR Template Preparation Kit. 1 × 10^6^ HT29 tumor cells were isolated by using the same kit in order to establish a standard curve for calibration of the DNA.

The extracted DNA was resuspended in 200 μl elution buffer and a sequential dilution series was prepared. To enable comparability the standard solutions that were previously prepared as a bulk preparation were diluted into murine DNA from untreated pfp/rag2 and rag2 mice.

### DNA extraction and real-time PCR for detection of circulating tumor cells

DNA extraction of murine blood was performed using the QIAamp DNA Blood Mini Kit and for DNA isolation of cell culture cells the QIAamp DNA Mini Kit (Qiagen, Hilden, Germany) was used, according to manufacturers′ instructions. Real-time polymerase chain reaction (PCR) and melting curve analyses were performed in glass capillaries with the Light Cycler 2.0 System. For the real-time PCR, the LightCycler Fast Start DNA MasterPLUS SYBR-Green I Kit (Roche Diagnostics GmbH, Mannheim, Germany) was used. Two μl of DNA solution was used as a template for the PCR reaction and incubated in a total reaction volume of 10 μl, containing 1x SYBR-Green I Master mix including Taq DNA polymerase, Taq PCR buffer, a dNTP mixture and 1 mmol/l MgCl2-, 10 pmol specific Alu primers. Forward Alu primer (TGG CTC ACG CCT GTA ATC CCA) and reverse Alu primer (GCC ACT ACG CCC GGC TAA TTT) were synthesized by MWG Biotech AG (Ebersberg, Germany). The PCR conditions were initially 10 min. 95°C, followed by 50 cycles of 5 s 95°C, 5 s 67°C and 20 s 72°C (measurement of fluorescence). Melting curve analysis (0 s 95°C, 12 s 65°C and 0 s 95°C) was performed directly after each PCR run [45]. To quantify circulating tumor cells a standard curve with 10 fold dilution of extracted DNA from 1×10^6^ cell culture cells HT29 was established. Control probes were isolated from mouse blood without inoculated tumor cells. Human tumor cells were quantified by real-time polymerase chain reaction (PCR) using established primers specific for the human alu sequences as previous described. For each sample, analyses were performed in triplicates and performed as independent experiments at least twice.

### Immunohistochemistry

The assessment of cell proliferation in the tumor population was made by a Feulgen stain of primary tumors performed as previously described [46].

### Quantitative methods and statistics

The percentage of cells showing mitotic figures in ten different areas of the tumor, delineated by an eyepiece graticule (310 μm^2^), was determined by counting a minimum of 500 cells from each animal. The areas of measurement were standardized: one corner of the eyepiece graticule was positioned at the tumor-host interface with an objective lens of magnification 10 and counting of the mitotic figures was carried out at the same site using an objective lens of magnification 400. Only cells that were in easily recognizable meta- and anaphases were counted as mitotic.

All values are presented as mean values. Statistically significant differences between both samples were calculated by a Mann–Whitney U test. Graph Pad Prism 6.0 (Intuitive Software for Science, San Diego, CA, USA) was used for statistical calculations. Differences were considered significant at p <0.05.

### Simulating the cancer spread

Computer simulations of cancer spread were performed in order to identify parts of the metastatic progress most influenced by NK cells. A previously developed computer model uses a discrete event simulation approach to analyze the metastatic progression [30, 31].

The main components of the computer model are so called compartments. They describe all parts that contain malignant cells such as the primary tumor, blood and distant metastases. The primary tumor and metastases are modelled as “continuous” compartments utilizing mathematical functions to describe the growth and spreading behavior of the tumor.

The growth of the primary tumor and metastases is modelled by a Gompertzian growth function that describes a sigmoid course:
1

The function *x*(*t*) provides the number of cells in the tumor at the time *t*. The parameter *a* represents the growth rate constant while *b* represents the size of the tumor at its saturated level. The parameter *t*_*0*_ allows to comprise a start size of the tumor, e.g. if the primary tumor starts as a single cell *t*_*0*_ will be 0. If the tumor starts as a cluster of cells, due to the injection of tumor cells into the mouse, *t*_*0*_ can be parameterized to display the size of the cluster. If a start size is given, *t*_*0*_ is automatically computed via an inverse function by the simulation software.

In this work it was assumed that 10^4^ cells of the injected one million tumor cells survived in the mice to form the primary tumor. Simulations with 10^3^ and 10^5^ cells were equally performed, but since the results do not differ significantly only results for 10^4^ cells are shown.

For the maximum tumor size *b* a value of 4.5 g was assumed. This value was estimated based on the experimental data. The value of the primary tumor growth rate constant *a* was computed using the determined mean values for the primary tumor weight and the duration of the experiment. The values are presented in the Results section.

The spread of metastases is described by the colonization rate *β*(*x*):
2

where *x* is the number of cells in the tumor, *m* is the colonization constant and *α* is the fractal dimension of blood vessels infiltrating the tumor which describes how well the tumor is supplied with blood. This value was assumed to be 0.663 which describes a superficial vascularity of the primary tumor [47]. This seems plausible since the primary tumor grows very fast. The colonization constant *m* was derived from the experimental data and is presented in the Results section.

To save computation time the colonization rate *β*(*x*) models only those malignant cells that eventually are able to establish a new metastasis. The so described cells can be killed by NK cells in the model. All disseminated tumor cells that die due to other factors than NK cells, either in the blood stream or in distant tissue are not displayed by *β*(*x*).

The blood is modelled as a “discrete” compartment where the behavior of cells is described employing events only. An event describes what happens in a compartment at a specific time, e.g. intravasation, apoptosis or extravasation. The probability with which different types of events occur can be parameterized for each discrete compartment.

Starting with the primary tumor that grows according to the function *x*(*t*), the first intravasation event is created conforming to the colonization rate *β*(*x*). After processing the intravasation event a new event is created which describes what happens next to the cell in the blood compartment, e.g. get killed by NK cells or extravasate and create a new metastasis. The probability of NK cell induced cell death in the blood compartment was determined based on the experimental data, as described in the Results section. Furthermore, the next intravasation event for the primary tumor is created conforming to the colonization rate *β*(*x*).

New metastases also grow according to the function *x*(*t*). Three different growth rate constants were considered for the lung metastases: 1/3, 1/2 and the same growth rate constant as the primary tumor. Metastases from metastases were neglected in the simulations, since the metastases are too small to be able to spawn metastases by themselves in the short duration of the experiment.

To determine why the metastases in pfp/rag2 and rag2 display the observed difference in size a potential dormancy of 21 and 30 days with a standard deviation of 7 days of the lung metastases was simulated. For modelling details on dormancy see next section.

A snapshot of the simulated system, containing the actual time, the size of the primary tumor, the number of metastases, the number of cells in all metastases and a size histogram of all metastases, is saved at a parameterizable interval. After the simulation covered a determined time span it will stop. Each configuration is simulated 100 times. Afterwards the mean and the standard deviation are computed.

A detailed description of the simulation process can be found in [30] and [31].

### Simulating dormancy and late dormancy

To simulate dormancy an extended version of the growth function stated in equation (1) was introduced to the computer model:
3

The parameter *t*_*d*_ and *t*_*ld*_ allow comprising dormancy and late dormancy [48–50] into the simulation. They display the duration of the dormancy phases. The duration can be parameterized with mean and standard deviation.

When a new metastasis is created which undergoes a dormancy phase, its status is set to “dormant” and the exact duration of the dormancy *t*_*d*_ is computed based on the stated mean and standard deviation. As long as the metastasis is in dormant state the simulation software will return a value of 1, whenever the size of the metastasis is enquired. As soon as the computed duration of the dormancy elapsed, the status of the metastasis is reset. The metastasis will now start growing conforming to the growth function stated in equation (3). The parameter *t*_*d*_ represents the offset between the creation of the metastasis and the time point when it starts to grow.

When a new metastasis is created which undergoes a late dormancy, the first step is to compute the size at which the metastasis passes into the late dormancy phase. The size is computed based on a parameterizable mean and standard deviation. The time point when the metastasis reaches the computed size is computed via an inverse of the growth function. Until this time point the metastasis will grow unrestricted conforming to the growth function stated in equation (3) with a value of 0 for the parameter *t*_*ld*_. Similar to normal dormancy its status is then changed into dormant state and the duration of the late dormancy phase *t*_*ld*_ is computed based on the stated mean and standard deviation. The simulation software will return the computed size whenever the size of the metastasis is enquired until the late dormancy phase elapsed. After the status of the metastasis is reset it will continue growing conforming to the growth function equation (3), but with an updated value for *t*_*ld*_ to include the accrued offset.

## Abbreviations

NK cells: Natural killer cells
pfp/rag2: Pore forming protein and recombination activating gene 2 double knock out
rag2: Recombination activating gene 2 only knock out
CTC: Circulating tumor cells
DTC: Disseminated tumor cells.
